# Idiopathic granulomatous orchitis: A case study

**DOI:** 10.1016/j.eucr.2024.102754

**Published:** 2024-05-14

**Authors:** Miguel Armando Benavides-Huerto, Jaime García-Figueroa, Venice Chávez-Valencia, Francisco Alejandro Lagunas-Rangel

**Affiliations:** a Laboratory of Pathology and Cytopathology “Dr. Miguel Benavides”, Morelia, Michoacán, Mexico; b High Specialty Medical Center, Moroleón, Guanajuato, Mexico; c Department of Nephrology. Hospital General Regional No.1. Instituto Mexicano del Seguro Social, Morelia, Michoacán, Mexico; d Department of Surgical Sciences, Functional Pharmacology and Neuroscience, Uppsala University, Uppsala, Sweden

**Keywords:** IGO, Inflammation, Orchiectomy, Autoimmune disease

## Abstract

Idiopathic granulomatous orchitis (IGO) is a rare inflammatory disorder of unknown etiology affecting the testis. Presented here is the case of a young patient who developed IGO, potentially associated with an anti-sperm antibody-mediated autoimmune response.

## 1 Introduction

Idiopathic granulomatous orchitis (IGO) is a rare inflammatory disorder affecting the testes.^1^ This disease is characterized by a non-caseating granulomatous inflammation of unknown etiology.^2^ Currently, there are only about 30 reports of this pathology in the literature, indicating its rarity and limited documentation. We present the case of a young Mexican patient who developed IGO, possibly associated with an autoimmune response mediated by antisperm antibodies.

## 2 Case report

**Fig. 1:**
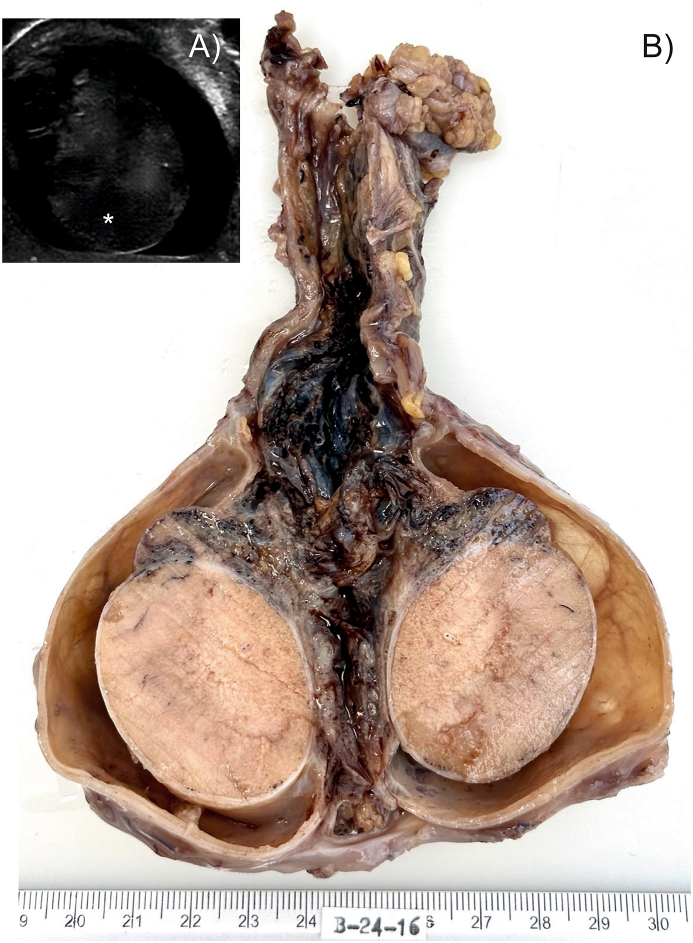
**Macroscopic findings**. A) Testicular ultrasound reveals areas of decreased echogenicity, highlighted with an asterisk for clarity. B) The illustration shows the result of the orchiectomy performed on the patient in this case.

A 22-year-old male presents to his physician because of persistent unilateral testicular pain and enlargement of the right testicle for three months. He denies any recent testicular trauma or previous genitourinary infections, and also reports no fever, chills or urinary symptoms. The patient, an office worker, also denies playing contact sports, having recently received vaccinations, or having had any recent illness, except for a common cold 5 months ago. He mentioned being sexually active with a stable partner and a child. During the physical examination, palpation confirms the enlargement of the right testicle, which is tender and firm. There are no signs of infection. The contralateral testicle appears normal in size and without palpable abnormalities. Transillumination of the scrotum indicates translucency, suggesting fluid accumulation within the scrotal sac. A testicular ultrasound confirmed the presence of fluid around the affected testicle, as well as revealed thickened testicular tissue with areas of decreased echogenicity (Fig. 1A). Sperm banking was carried out for possible future family planning. Due to the high suspicion of malignancy and in response to an acute and intense episode of pain, a radical orchiectomy was performed.

The extracted testicle, measuring 4 x 3.3 × 3.6 cm, was ovoid and turgid. On dissection, its interior revealed a velvety texture with a homogeneous region clearly delimited by a 2 mm thick albuginea, smooth to the touch. Around the testis, the tunica vaginalis is dilated and rigid, with a thickness varying from 1 mm to 3 mm and a polished surface. Between the albuginea and the tunica vaginalis there is a wide space filled with citrine fluid. The rete testis was spongy and purplish-gray in color. In addition, the spermatic cord, of irregular tubular shape and smooth, rubbery texture, measures 9 x 2 × 2 cm. Its gray exterior, when sectioned, reveals a yellowish-gray cavernous interior (Fig. 1B).

**Fig. 2:**
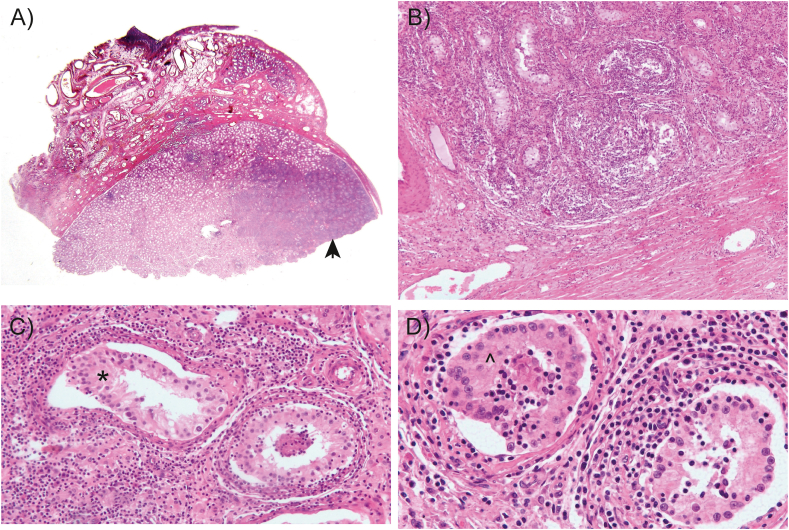
**Histological findings.** A) The testicular parenchyma on the left had a normal appearance, while the right side showed intense inflammation (arrow) (H&E, 1:1). B) The inflammation produced a darkening of the tubules, with hyperplastic mesothelium in the albuginea (H&E, 2.5X). C) The inflammation did not affect the germinal epithelium (asterisk) (H&E, 10X). D) Although the inflammatory infiltrate accumulated in the lumen of the tubules (caret), no evidence of necrosis was observed (H&E, 20X).

The pathology report revealed that the architecture of the seminiferous tubules within the testicular parenchyma was well preserved, maintaining their structural integrity. However, the interstitial space, which lies between these tubules, showed a dense inflammatory response (Fig. 2A). This response was composed mainly of lymphocytes, accompanied by a smaller proportion of plasma cells, and interspersed with a small number of epithelioid macrophages. Notably, the inflammation was so widespread that it obscured the Leydig cells located around the seminiferous tubules (Fig. 2B), suggesting an aggressive inflammatory process. Closer examination of the tubular basement membranes showed that they are preserved but thickened, indicating a chronic condition. The germinal epithelium was well differentiated, but showed areas of arrested maturation, a sign of interrupted spermatogenesis (Fig. 2C). Within the lumina of the tubules there was an accumulation of neutrophils, epithelioid histiocytes and a lymphoplasmacytic inflammatory infiltrate, although there was no evidence of necrosis (Fig. 2D). The tunica vaginalis, the outer covering of the testis, also showed signs of inflammation, characterized by a predominantly perivascular and interstitial mononuclear inflammatory reaction. In addition, the surface of the tunica vaginalis showed mesothelial hyperplasia, indicative of a reactive process to the ongoing inflammation. Meanwhile, the rete testis maintained a normal histologic appearance, unaffected by the inflammatory and reactive changes observed in the surrounding structures. However, within the spermatic cord, the blood vessels of the pampiniform plexus were markedly congested, dilated and tortuous, and the surrounding interstitial space appeared edematous, further emphasizing the systemic nature of the inflammatory response affecting the testicular environment.

Investigations were carried out to identify possible causes such as tuberculosis, sifilis, actinomycosis and sarcoidosis, which could explain the observed inflammatory reaction. Unfortunately, the results of these tests did not reveal any evidence to support the presence of these conditions. However, the patient's serum showed low titers of antisperm antibodies, indicating a possible autoimmune etiology.

Based on the comprehensive findings outlined, the diagnosis of IGO was conclusively established, concurrent with the presence of a hydrocele. The patient was discharged after three days and has since been followed up for a period of 10 months. During this time, he has had no complaints or abnormal physical findings.

## 3 Discussion

Although IGO can occur over a wide age range (19–84 years), it most commonly manifests between the ages of 50 and 70 years.^2^ Bilateral testicular involvement is relatively rare.^3^ The case presented here is noteworthy, as the patient was notably younger (22 years), representing an atypical incidence within this age range.^4^ The typical presentation of the chronic form of IGO consists of a unilateral scrotal mass that increases in size, with or without chronic pain. However, in the less common acute form, patients experience sudden moderate to severe pain, sometimes accompanied by other symptoms such as fever, hematuria, dysuria and hydrocele.^2^^,^^4^ Our patient also presented a hydrocele accompanying IGO.

No definitive etiologic factor for IGO has been identified. However, external or iatrogenic trauma has been proposed as a significant contributing factor, which could trigger a granulomatous reaction by allowing extravasation of sperm into the testis. In addition, autoimmune responses, particularly sperm-targeted autoantibodies, have been identified as another possible cause.^2^ In the case presented, antisperm antibodies were identified in low titles, suggesting a possible association with the development of IGO in the patient. Associations with certain genitourinary infections have also been established in the development of IGO.^1^

In many cases, this pathology is initially confused with testicular tumors, such as seminoma or lymphoma cell infiltration, and physical examination often fails to distinguish between benign and malignant conditions.^2^ Typical findings of IGO on ultrasound include diffusely hypoechoic, poorly defined, or well-defined focal intratesticular areas.^1^ Thickening of the epididymis, scrotal skin, or tunica albuginea may indicate a benign inflammatory process on ultrasound. Nevertheless, differentiating tissue destruction and fibrosis resulting from intratesticular processes from testicular neoplasia remains a significant challenge.^5^ Infectious granulomatous orchitis represents another important consideration in the field of differential diagnoses. It includes conditions such as tuberculosis, syphilis, brucellosis and lepromatous leprosy, as well as sarcoidosis.^2^

In the chronic form of IGO, conservative therapy with antibiotics, steroids and anti-inflammatory agents has proven ineffective. Notably, the definitive treatment remains orchiectomy.^3^^,^^4^ Although some studies have highlighted the potential use of biopsies and a conservative approach, these strategies have not gained wide acceptance.^1^^,^^2^ Other research supports orchiectomy, citing the extensive damage to all testicular tissue from the inflammatory process at presentation.^2^

## Ethical approval

Not applicable.

## Informed consent statement

Written informed consent has been obtained from the patient to publish this paper.

## Competing interests

The authors declare no conflict of interest.

## Funding

This research did not receive funding from any specific grant provided by public, commercial, or not-for-profit sectors. The open access publication fee is covered by Uppsala University through the BIBSAM agreement.

## Availability of data and materials

Data sharing is not applicable to this article as no new data were created or analyzed in this study.

## Acknowledgements

No acknowledgments.

## CRediT authorship contribution statement

**Miguel Armando Benavides-Huerto:** Writing – review & editing, Investigation, Formal analysis, Conceptualization. **Jaime García-Figueroa:** Methodology. **Venice Chávez-Valencia:** Writing – review & editing. **Francisco Alejandro Lagunas-Rangel:** Writing – review & editing, Writing – original draft, Resources, Investigation, Formal analysis, Data curation, Conceptualization.
